# Editorial: Petal Development: From Cell Biology to EvoDevo

**DOI:** 10.3389/fpls.2022.951442

**Published:** 2022-06-15

**Authors:** Tengbo Huang, Elena M. Kramer, Deshu Lin

**Affiliations:** ^1^Guangdong Provincial Key Laboratory for Plant Epigenetics, College of Life Sciences and Oceanography, Shenzhen University, Shenzhen, China; ^2^Department of Organismic and Evolutionary Biology, Harvard University, Cambridge, MA, United States; ^3^Key Laboratory of Ministry of Education for Genetics, Breeding and Multiple Utilization of Crops, Fujian Agriculture and Forestry University, Fuzhou, China

**Keywords:** petal organogenesis, cell proliferation and expansion, cell type specification, plant-pollinator interaction, plant evolution

The petal is an excellent model system for studying plant organogenesis because it is simple in structure and dispensable for plant viability (Irish, 2008). The petal is also a useful system for investigating plant-animal interactions, as it is the major plant part that attracts pollinators in many outcrossing species (Fenster et al., 2004; Stuurman et al., 2004). The nine articles in this Research Topic focus on several important aspects of research associated with petal development and function. We organize this collection of studies into several groups based on common themes as described below.

## Specification of Petal Identity

In the model plant *Arabidopsis thaliana* (Arabidopsis, Brassicaceae), petal primordia arise in the second whorl of the four concentric floral whorls. Specification of petal identity depends on the combinatorial regulation of MADS box transcription factors encoded by the *APETALA1* (*AP1*), *APETALA3* (*AP3*), *PISTILLATA* (*PI*), and *SEPALLATA* (*SEP*) genes (Wellmer et al., 2014). In this Research Topic, Deveaux et al. investigate an ortholog of *AP3, NdAP3-3*, in *Nigella damascena* (Ranunculaceae). They analyze the putative target genes of *NdAP3-3* and compare them with those of the *AP3* homologs in Arabidopsis and *Aquilegia coerulea* (Ranunculaceae). They identify a number of common genes in each comparison, which shows the functional conservation of *AP3* genes during plant evolution.

## Petal Growth Mediated by the Cell Proliferation to Expansion Transition

The growth of petal primordia in the second whorl occurs through two waves:early stage cell division and late stage post-mitotic expansion (Huang and Irish, 2016). Coordination of cell division and expansion in petal development is critical for the regulation of cell number and size that determine the morphology of the mature petal. In this Research Topic, several articles focus on studies related to this theme. For example, Galipot et al. investigate the growth of nectariferous petals in *N. damascena* and identify the allometric nature of petal shape and size dynamics. They also find that the patterning of petal shape is primarily determined by the activities of cell proliferation, while the change of petal size is mainly driven by the dynamics of cell expansion. These data uncover an important cellular mechanism that sculpts the elaborate *N. damascena* petal. Furthermore, in a study of another ornamental species, *Gerbera hybrida* (Asteraceae), Lin et al. characterize two 14-3-3 protein-coding genes that mediate brassinosteroid-induced ray petal elongation. They find that these two 14-3-3 genes play a key role in controling petal growth by promoting petal cell elongation.

The timing of transition from cell proliferation to expansion is a key control point that determines mature organ morphology. Two transcription factors, RABBIT EARS (RBE) and TCP5, play vital roles in the regulation of cell proliferation-to-expansion transition in the Arabidopsis petal (Huang and Irish, 2015). In this Research Topic, Huang et al. propose a model that RBE may repress *TCP5* by recruiting epigenetic modifiers to induce chromatin-mediated silencing at the *TCP5* locus, which adds a new layer of epigenetic regulation to the RBE/TCP5-mediated transcriptional control of cell proliferation-to-expansion transition in petal morphogenesis.

## Regulation of Petal Cell Maturation

Following the transition from cell division to expansion, another critical factor that controls petal growth is the degree of expansion and final cell shape. Zhang and Zhang show that genome size of *Paphiopedilum* (Orchidaceae) species is negatively correlated with labellum epidermal cell size, and positively correlated with the size of leaf epidermal cells, which suggests that genome size may be a strong predictor of cell size. Moreover, Cavallini-Speisser et al. write a review to summarize the major cell types in the petal and their functions. They also conclude the actions of the key regulators of petal development in the specification of different cell types in the petal. This collection of information provides us with a good resource for further studying the genetic control of petal organogenesis at the cellular level.

## Relationship Between Petal Development and Pollination

Variation in petal development that results in the diversity of petal morphologies has important implications in attracting specific pollinators for different plant species (Fenster et al., 2004; Stuurman et al., 2004). One such relationship between petal development and pollination is investigated by Hsu and Kuo, who analyze the nectar guide patterns in Ligeriinae (Gesneriaceae) by examining the petal contours and vasculatures. They identify four modes of nectar guide patterns and show that two of the four modes, distal and proximal, have the strongest associations with pollination types. These results are helpful for further understanding the diversity of nectar guide patterns and its relationship with plant-pollinator interaction in Ligeriinae.

## Summary

The diverse and interconnected subjects presented in this Research Topic provide comprehensive information about ongoing studies of petal development and functions. These studies also raise some interesting questions worthy of future investigation. For example, what role do epigenetic mechanisms play in the transcriptional regulation of petal growth? How is variation in growth patterns during petal development, which has been shown in this topic to be critical for determining petal morphology, regulated by temporal- and spatial-specific factors? It is worth noting that we include two articles that are not directly related with petal development in this topic: one uses tissue-specific analyses to investigate the interaction between tomato and a parasitic plant *Cuscuta campestris* (Jhu et al.); while the other assesses the miRNA profiles in Mepiquat chloride-mediated inhibition of internode elongation in cotton (Wang et al.). The strategies and results of these two studies, such as the tissue-specific approaches and the roles of the epigenetic regulator miRNAs, may shed light on the above questions. By addressing these critical questions, we hope to have a more in-depth dissection of the complex network that controls petal growth, which may serve as the basis for a better understanding of plant organ development in the future.

## Author Contributions

TH, EK, and DL were contributed to the writing of this editorial. All authors approved it for publication.

## Funding

Our work on petal organogenesis has been supported by grants from the Guangdong Basic and Applied Basic Research Foundation (2021A1515011035) and Guangdong Special Support Program for Young Talents in Innovation Research of Science and Technology (2019TQ05N940) to TH, and NSF IOS- 1456217 to EK.

## Conflict of Interest

The authors declare that the research was conducted in the absence of any commercial or financial relationships that could be construed as a potential conflict of interest.

## Publisher's Note

All claims expressed in this article are solely those of the authors and do not necessarily represent those of their affiliated organizations, or those of the publisher, the editors and the reviewers. Any product that may be evaluated in this article, or claim that may be made by its manufacturer, is not guaranteed or endorsed by the publisher.

## References

[B1] Fenster C. B. Armbruster W. S. Wilson P. Dudash M. R. Thomson J. D. (2004). Pollination syndromes and floral specialization. Annu. Rev. Ecol. Evol. Syst. 35, 375–403. 10.1146/annurev.ecolsys.34.011802.132347

[B2] Huang T. Irish V. F. (2015). Temporal control of plant organ growth by TCP transcription factors. Curr. Biol. 25, 1765–1770. 10.1016/j.cub.2015.05.024 26073137

[B3] Huang T. Irish V. F. (2016). Gene networks controlling petal organogenesis. J. Exp. Bot. 67, 61–68. 10.1093/jxb/erv444 26428062

[B4] Irish V. F.. (2008). The Arabidopsis petal: a model for plant organogenesis. Trends Plant Sci. 13, 430–436. 10.1016/j.tplants.2008.05.006 18603466

[B5] Stuurman J. Hoballah M. E. Broger L. Moore J. Basten C. Kuhlemeier C. (2004). Dissection of floral pollination syndromes in Petunia. Genetics 168, 1585–1599. 10.1534/genetics.104.031138 15579709 PMC1448759

[B6] Wellmer F. Graciet E. Riechmann J. L. (2014). Specification of floral organs in Arabidopsis. J. Exp. Bot. 65, 1–9. 10.1093/jxb/ert385 24277279

